# Supplementary figures and images for: Vast cryptic diversity in direct-developing frogs Pristimantis (Anura: Strabomantidae): a new subgenus and the description of a new species from the eastern Andes of Ecuador
Source: PeerJ. 2025 Dec 17;13:e20512. doi: 10.7717/peerj.20512 (PMC12717849; doi:10.7717/peerj.20512)

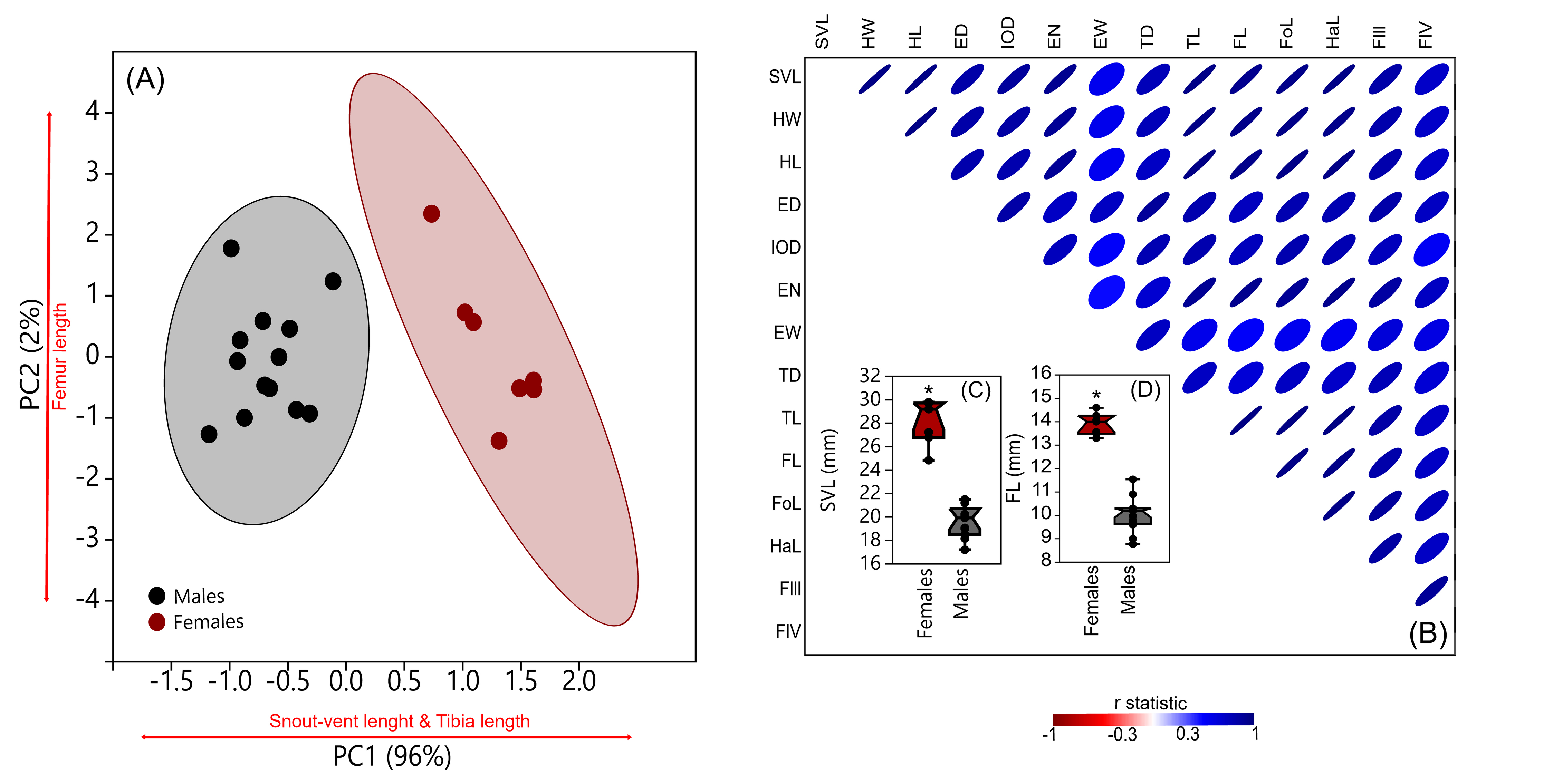

Supplement: Supplemental Information 2 — (A) Principal component analysis, with Snout vent length (SVL), Tibia length (TL) and Femur length (FL) as variables which most explain data variance (PC1 and PC2=98%); ellipsoids represent 95% of confidence limits; (B) Correlogram of morphometric measurements with Linear r statistics represented as colored ellipsoids; Boxplot comparisons of (C) Snout vent length (SVL) and (D) Femur length (FL) of males and females; asterisk represent statistical differences inferred by Mann-Whitney U test. [file peerj-13-20512-s002.jpg]
