# Supplementary material for: Vast cryptic diversity in direct-developing frogs Pristimantis (Anura: Strabomantidae): a new subgenus and the description of a new species from the eastern Andes of Ecuador
Source: PeerJ. 2025 Dec 17;13:e20512. doi: 10.7717/peerj.20512 (PMC12717849; doi:10.7717/peerj.20512)

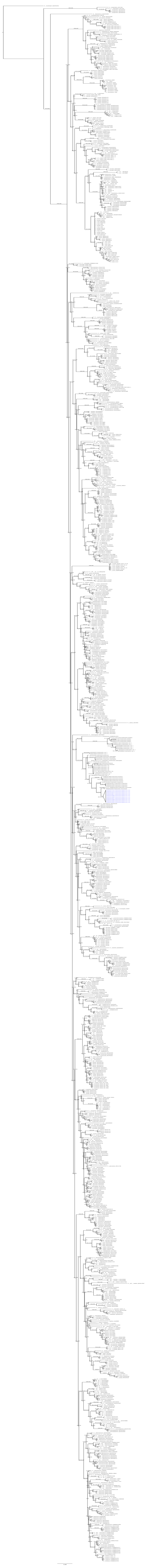

Supplement: Supplemental Information 1 — Phylogenetic relationships of the genus Pristimantis, with the position of the Cryptomatis subgenus. [file peerj-13-20512-s001.pdf]
